# Longitudinal Association Between Visual Function and Physical Performance Among Older Adults: Findings From NHATS

**DOI:** 10.1093/geroni/igaf122.076

**Published:** 2025-12-31

**Authors:** Shu Xu, Joshua Ehrlich, Mengyao Hu

**Affiliations:** University of Michigan, Ann Arbor, Michigan, United States; University of Michigan, Ann Arbor, Michigan, United States; The University of Texas Health Science Center, Houston, Texas, United States

## Abstract

Older adults with a visual impairment (VI) are more likely to experience mobility limitations; however, few studies have examined how changes in specific visual functions influence mobility trajectories. This study investigates the longitudinal association between visual function and physical performance among U.S. older adults. We analyzed three-year longitudinal data (2021-2023) from Medicare beneficiaries ≥71 years in the 2021-2023 National Health and Aging Trends Study. Visual function was assessed based on presenting binocular distance visual acuity [DVA] and contrast sensitivity [CS]. Any VI was defined as either distance VI [>0.30 logMAR] or CS impairment [<1.55 logCS]. Physical performance was measured using Short Physical Performance Battery (SPPB; range: 0–12), with higher scores indicating better physical performance. Growth curve modeling was used to analyze longitudinal associations. Among 2,707 participants, 31.3% had any VI. Older adults with any VI had lower SPPB scores than those without any VI (mean: 6.01 vs. 7.70). Growth curve models showed significant between-person effects for both DVA (B = -3.24, p <.001) and CS (B = -2.99, p <.001), as well as significant within-person effects for DVA (B = -0.65, p <.01) and CS (B = -0.51, p <.01). In conclusion, older US adults with worse overall visual function have lower physical performance, and declines in visual function over time are associated with faster declines in physical performance. Findings highlight the critical role of vision health in maintaining mobility in later life.

